# Dust and Its Dangers

**Published:** 1891-05

**Authors:** 


DUST AND ITS DANGERS. By T. MITCHELL PRUDDEN, M. D. Author of "A Manual of Practical Histology," "The Story of the Bacteria," etc., Illustrated. 16mo, cloth. P. 111. G. P. Putnam's Sons, New York, 27 W. Twenty-third St. London, 27 King William Street Strand. 1890. Price, 75c.

Dr. Prudden handles this subject very expertly, and shows that this omni-present article may be an important factor in the transmission of disease. The Presence of Microörganisms in Dust is virtually his text; and the dangers thereof is made manifestly apparent. It is an attractive and at the same time useful little work, and could be read with much benefit by the masses.

